# Support Materials of Organic and Inorganic Origin as Platforms for Horseradish Peroxidase Immobilization: Comparison Study for High Stability and Activity Recovery

**DOI:** 10.3390/molecules29030710

**Published:** 2024-02-03

**Authors:** Muhammad Bilal, Oliwia Degorska, Daria Szada, Agnieszka Rybarczyk, Agata Zdarta, Michal Kaplon, Jakub Zdarta, Teofil Jesionowski

**Affiliations:** 1 Department of Sanitary Engineering, Faculty of Civil and Environmental Engineering, Gdansk University of Technology, G. Narutowicza 11/12, PL-80233 Gdansk, Poland; 2 Advanced Materials Center, Gdansk University of Technology, 11/12 Narutowicza, PL-80233 Gdansk, Poland; 3 Institute of Chemical Technology and Engineering, Faculty of Chemical Technology, Poznan University of Technology, Berdychowo 4, PL-60965 Poznan, Poland; oliwia.degorska@gmail.com (O.D.); daria.szada@put.poznan.pl (D.S.); agata.zdarta@put.poznan.pl (A.Z.); michal.kaplon@student.put.poznan.pl (M.K.); teofil.jesionowski@put.poznan.pl (T.J.)

**Keywords:** nanomaterials, support material, horseradish peroxidase, immobilization, catalytic activity, enzyme stability

## Abstract

In the presented study, a variety of hybrid and single nanomaterials of various origins were tested as novel platforms for horseradish peroxidase immobilization. A thorough characterization was performed to establish the suitability of the support materials for immobilization, as well as the activity and stability retention of the biocatalysts, which were analyzed and discussed. The physicochemical characterization of the obtained systems proved successful enzyme deposition on all the presented materials. The immobilization of horseradish peroxidase on all the tested supports occurred with an efficiency above 70%. However, for multi-walled carbon nanotubes and hybrids made of chitosan, magnetic nanoparticles, and selenium ions, it reached up to 90%. For these materials, the immobilization yield exceeded 80%, resulting in high amounts of immobilized enzymes. The produced system showed the same optimal pH and temperature conditions as free enzymes; however, over a wider range of conditions, the immobilized enzymes showed activity of over 50%. Finally, a reusability study and storage stability tests showed that horseradish peroxidase immobilized on a hybrid made of chitosan, magnetic nanoparticles, and selenium ions retained around 80% of its initial activity after 10 repeated catalytic cycles and after 20 days of storage. Of all the tested materials, the most favorable for immobilization was the above-mentioned chitosan-based hybrid material. The selenium additive present in the discussed material gives it supplementary properties that increase the immobilization yield of the enzyme and improve enzyme stability. The obtained results confirm the applicability of these nanomaterials as useful platforms for enzyme immobilization in the contemplation of the structural stability of an enzyme and the high catalytic activity of fabricated biocatalysts.

## 1. Introduction

Science and industry have become increasingly interested in enzymatic biocatalysts due to their unique characteristics, including their robustness, selectivity, specificity, and efficient kinetics. Furthermore, enzymatic catalysts are characterized by low energy and equipment requirements and are known for their environmentally friendly characteristics [1,2]. Commercializing the use of enzymes meets several challenges, primarily in terms of economic considerations and the difficulty of reusing free enzymes. It is important to note that biocatalysts often have limited structural stability in varying reaction media and conditions [3]. The immobilization of enzymes can improve their properties by combining them with an insoluble carrier, resulting in heterogeneous biocatalysts. Immobilization also enhances the thermal and storage stability of enzymes and enables them to maintain their activity over a wide pH range in aqueous solutions, as well as in organic solvents. Further, immobilized enzymes can be quickly and easily isolated from the reaction medium, significantly improving their reusability and reducing costs [4,5]. As a result of immobilization, efficient biocatalytic systems can be produced that combine both an enzyme’s advantages as well as the physical properties of the selected carrier [6].

A prerequisite for the proper execution of the immobilization process is the selection of a suitable carrier. Substances used as carriers should be inert compounds characterized by high chemical and thermal stability [7]. Over the past years, the development of nanotechnology has provided access to diverse nanomaterials, which, due to their unique properties, have created prospects for use as nanocarriers in enzyme immobilization [8,9]. The availability of various forms of nanomaterials, from branched dendrimers and matrices through fibers and particles to tubes and dots, allows for the selection of a matrix with specific characteristics tailored to the properties of the biocatalysts, resulting in an easier immobilization protocol [10]. Compared to conventional matrices, nanocarriers feature an ease of surface modification, enhanced stability, and reusability [11,12]. Metallic nanoparticles have gained particular recognition due to their high surface-to-volume ratio, strength, reactivity, and chemical inertness [13,14]. One of the examples may be iron oxide nanoparticles, further distinguished by their superparamagnetic properties and biocompatibility, which were discussed by Mohamed et al. [15] to immobilize horseradish peroxidase (HRP). The produced system was characterized by a significant improvement in the stability of the enzyme and the ability to easily separate the product from the medium by magnetic separation, which translated into the preservation of more than 55% of its initial activity after 10 reaction cycles. Titanium dioxide nanoparticles exhibiting UV resistance and photocatalytic properties have also been successfully used in enzyme immobilization. Sahin [16], for instance, used 3-(3,4-dihydroxyphenyl) propionic acid-functionalized TiO_2_ nanoparticles as matrices for HRP immobilization. Given their abundant availability and polymeric nature, the use of non-metallic nanoparticles, including biodegradable chitosan or thermally stable silica, has also gained popularity [17]. Melo et al. [18] combined chitosan nanoparticles with polyethylene glycol (PEG) for horseradish peroxidase immobilization. The steps carried out improved biocompatibility and preserved enzyme activity by up to 70%, thus representing promising biocatalytic systems for biomimetic applications. The enormous potential of nanobiocatalysts makes them applicable in the chemical, pharmaceutical, or medical industries, as well as in the production of biofuels, the degradation of harmful substances, and many others [19,20].

Horseradish peroxidase (HRP) is a widely distributed enzyme with well-researched properties and is one of the most influential enzymes in biotechnology. Due to its extensive catalytic activity and wide substrate range, it is capable of carrying out oxidation reactions of a wide variety of organic and inorganic compounds in reactions supported by hydrogen peroxide [21,22]. As a result, HRP is widely used in many applications. Due to these features, biocatalytic systems based on HRP immobilized on nanocarriers are of particular interest [23]. Essential HRP applications include the design of advanced biosensors that detect hydrogen peroxide. For example, Devaraj et al. [24] used graphene oxide–chitosan gold nanocomposites as electrode platforms for the immobilization of HRP to investigate the second generation of enzymatic biosensors. A technique for developing an enzymatic biosensor based on HRP immobilized on single-walled carbon nanotubes treated with acid has been proposed by Sahin [25]. The expansion of the global industry has resulted in a significant increase in the presence of a variety of pollutants in the environment that can negatively impact both human health and the environment. Biocatalytic systems based on HRP immobilized onto nanomaterials have remarkable potential for purifying surface waters and wastewaters of a wide range of impurities, such as dyes, pharmaceuticals, pesticides, phenols, and everyday products [26,27,28,29]. A study by Aldhari et al. [26] demonstrated that HRP immobilized on copper oxide nanosheets is an efficient means of degrading a variety of dyes (crystal violet, methyl green, malachite green). On the other hand, Machałowski et al. [30] used silica nanopowder and 3D fibrous chitin scaffolds from the marine sponge *Aplysina fistularis* as carriers for HRP immobilization, and the implemented biosystem catalyzed almost the 100% degradation of 17α-ethinylestradiol.

The use of HRP offers a wide range of possibilities in the context of biocatalysis development. Developing new nanomaterials suitable for HRP immobilization is one of the greatest challenges that will enable the production of highly active biocatalytic systems for multipurpose applications. Nevertheless, the occurring research gaps related to the broad characterization of hybrid materials and biocatalytic systems, as well as their increased stability and activity, are not fully covered. Many studies show greater stability and the possibility of multiple applications of biocatalysts; nevertheless, the improvement in catalytic activity could be increased. There is also a lack of comprehensive studies on horseradish peroxidase immobilization on various support materials. Hence, in the presented study, it was attempted to obtain novel biocatalytic systems based on single or hybrid nanomaterials as supports for HRP immobilization to determine which material acts as a suitable platform for HRP immobilization with high activity and stability retention. The obtained systems were characterized in terms of their morphology, and their immobilization efficiency, biocatalytic activity, reusability, and storage stability were also determined. In the presented work, the broad characterization of the support materials and biocatalytic systems covers research gaps occurring in the literature, providing data about the use of novel materials with applications as immobilization platforms, as well as improving the stability and activity of the obtained biocatalytic systems.

## 2. Results and Discussion

### 2.1. Physicochemical Characterization of Materials before and after Immobilization

Due to a variety of functional groups occurring on the surface of the materials and their high surface capacity, inertness, and accessibility, zeolitic imidazole framework (ZIF-8), graphene oxide, MWCNT, MNP, and CH-M-Se materials were tested as immobilization platforms for the HRP enzyme to obtain biocatalysts with a broad spectrum of applications. It should be noted that each support material is characterized by slightly different chemical/physical phenomena, resulting in varying properties of the immobilized enzymes produced. For instance, the ZIF-8 material is known for its open 3D structure, which limits diffusional limitations and allows easy access to enzymes that might be deposited on the surface of this material into its pores. In contrast, the use of MWCNT or graphene oxide as a support results in an improvement in the transport of electrons between an enzyme and a substrate, which facilitates enzyme activity. These materials are also known for their well-developed surface area, which improves the even distribution of biomolecules. In contrast, MNPs were used due to their magnetic properties, which facilitate the separation of the biocatalysts from the reaction mixture and enhance their reusability. Finally, in the chitosan-coated magnetic nanoparticles doped with a selenium ion (Ch-M-Se) hybrid material, several features were combined to produce a synergistic effect on the enzyme’s properties. Chitosan offers biocompatibility as well as hydroxyl and amine functional groups for stable enzyme binding. Magnetic nanoparticles, as mentioned above, support system recovery, whereas the addition of selenium ions improves the activity of the immobilized HRP. A comprehensive characterization of the support materials used and the materials after HRP immobilization was performed in order to clearly examine the suitability of the hybrid materials for their use in immobilization, as well as to confirm effective enzyme binding and determine changes in the support materials after enzyme deposition.

**Figure 1:**
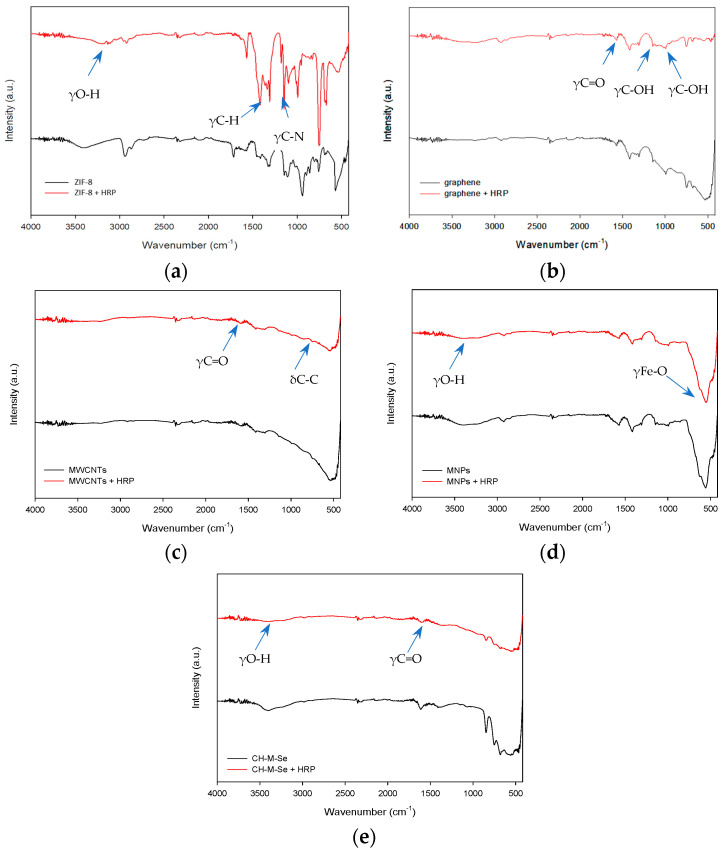
FTIR spectra of pristine hybrid materials and hybrid materials with immobilized enzyme: (**a**) ZIF-8 carrier before and after HRP immobilization; (**b**) graphene oxide material before and after HRP immobilization; (**c**) MWCNT material before and after HRP immobilization; (**d**) magnetic nanoparticles (MNPs) before and after HRP immobilization; and (**e**) CH-M-Se carrier before and after HRP immobilization.

At first, Fourier-transform infrared spectroscopy was applied. The Fourier-transform infrared spectroscopy (FTIR) spectra of the ZIF-8 sample (Figure 1a) show that the broad band visible around 3500–3000 cm^−1^ comes from the stretching vibrations of O-H groups occurring on the sample’s surface, allowing enzyme embedding. Additionally, bands from 2-methylimidazole are visible on the spectrum around 1150 cm^−1^ and 1180 cm^−1^ coming from the vibrations of the C–N bond, and the signal around 1310 cm^−1^ and 1420 cm^−1^ occurs from the vibration of the C–H bond, which corresponds with the obtained data from Xu et al. [31]. The graphene spectrum (Figure 1b) shows multiple signs of oxygen-derived functional groups occurring in the structure. For example, the band around 1600 cm^−1^ might occur from C=O in carboxylic COOH groups. The band around 1040 cm^−1^ refers to stretching vibrations from the C-O group, and the signal at 1375 cm^−1^ is characteristic of C-OH [32,33]. The FTIR spectrum of the multi-walled carbon nanotubes (MWCNTs, Figure 1c) shows a typical band around 600 cm^−1^, which corresponds with the bending vibrations of C-C bonds. The weak signal around 1730 cm^−1^ may be associated with the stretching vibrations of bonds from carboxylic groups, suggesting that carboxylic groups could be formed due to the oxidation of some carbon atoms on the surfaces of the MWCNTs [34].

For the magnetic nanoparticles (MNPs, Figure 1d), the FTIR spectrum consists of a band around 3440 cm^−1^ from the stretching vibrations of O-H groups occurring on the surface of the material. A typical band confirming the successful material’s synthesis appears around 584 cm^−1^, which originates from the stretching vibrations of Fe-O bonds [35]. For the last hybrid material tested, a broad band around 3500 cm^−1^ is visible, showing the presence of the stretching vibrations of O-H bonds occurring on the surface of the material. A typical band around 1740 cm^−1^ appears due to the stretching vibrations coming from the C=O bonds [36]. The functional groups of pristine materials, confirmed with FTIR spectroscopy, show the possibility of enzymes’ bonding to the surface of the support. The obtained spectra for pristine materials compared with the spectra of materials with immobilized enzymes do not significantly differ; nevertheless, the changes that occurred indicate the successful deposition of the biocatalyst on the support surfaces. For the materials after the immobilization process, the characteristic bands coming from vibrations of amide I, II, and III bonds are slightly visible around 1645, 1550, and 1250 cm^−1^, which are typical for the spectra of samples containing enzymes and confirm the successful immobilization [37]. Moreover, the broad band around 3500 cm^−1^ after immobilization is less intense in the analyzed samples, suggesting enzyme deposition mainly via hydroxyl groups.

**Figure 2:**
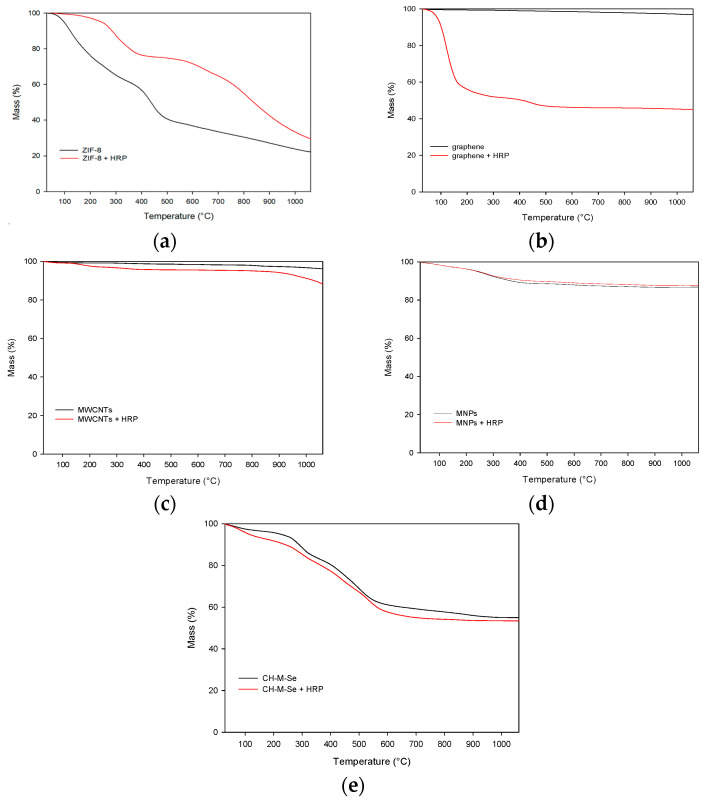
Results of thermogravimetric analysis of pristine hybrid materials and hybrid materials with immobilized enzyme: (**a**) pristine ZIF-8 carrier before and after HRP immobilization; (**b**) graphene oxide material before and after HRP immobilization; (**c**) pristine MWCNT material before and after HRP immobilization; (**d**) pristine MNPs before and after HRP immobilization; and (**e**) pristine CH-M-Se carrier before and after HRP immobilization.

In the next step, a thermogravimetric analysis was performed for all the materials (Figure 2). The obtained thermogravimetric analysis (TGA) curves of all the samples show a lack of great mass loss until 100 °C, which corresponds to the absence of entrapped water in pores. For the MWCNT and MNP samples, the mass loss for pristine materials and the obtained biocatalysts does not exceed 15%. This shows the great stability of the obtained materials and biocatalysts produced based on these materials. In contrast, the obtained TGA for the graphene-based biocatalysts shows a greater mass loss compared to the pristine materials due to the high enzyme loading and its degradation at high temperatures [38]. For the rest of the samples, the mass loss of the biocatalysts is comparable to that of the support materials, leading to the conclusion that the obtained biocatalysts are resistant to high temperatures and the mass of the enzyme loaded is lower; hence, the effect of the thermal degradation of the enzyme backbone on the mass of the sample is less pronounced. In comparison, the (3-aminopropyl)triethoxysilane (APTES) functionalized MNPs presented by Keshta et al. [38] display a mass loss of around 25% and 15% compared to the pristine and modified support materials, which correspond to the ones presented in the article. A greater mass loss at around 100 °C may be related to the physically adsorbed water present in the material. On the other hand, Song et al. [39] used ZIF-8 nanoparticles for enzyme deposition. The free HRP’s mass loss was greater at lower temperatures than that of the pristine support and the obtained biocatalyst. The results obtained confirmed the stabilizing effect of the support and its inertness at high temperatures.

**Figure 3:**
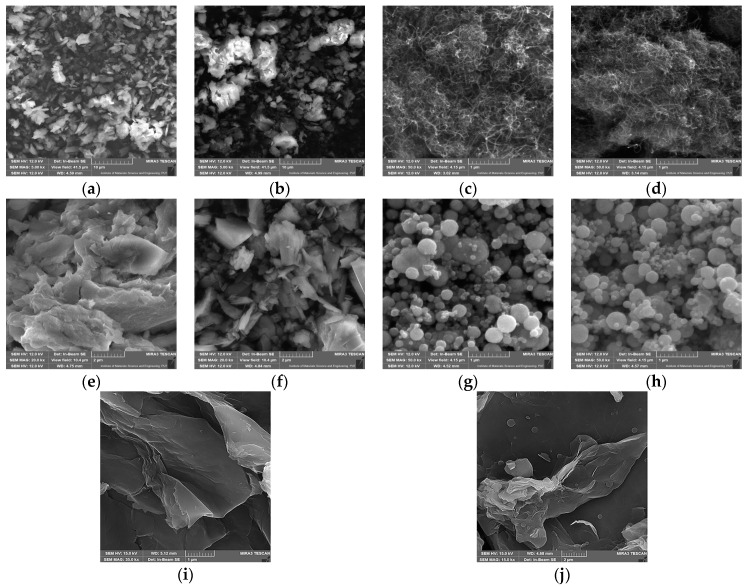
SEM images: (**a**) pristine ZIF-8 carrier; (**b**) ZIF-8 carrier after HRP immobilization; (**c**) pristine MWCNT material; (**d**) MWCNTs after HRP immobilization; (**e**) CH-M-Se carrier; (**f**) CH-M-Se carrier after HRP immobilization; (**g**) MNPs; (**h**) MNPs after HRP immobilization; (**i**) graphene oxide material; and (**j**) graphene oxide after HRP immobilization.

Both pristine materials and the obtained biocatalytic systems after HRP immobilization were also analyzed using scanning electron microscopy (SEM, Figure 3) and by determining changes in the particle size of the support before and after immobilization (Table 1). This allowed for an evaluation of the morphological structures of the applied carriers and a partial confirmation of the immobilization process. The resulting SEM images of all the materials are shown in Figure 3. Additionally, Table 1 shows the average particle size and the particle size distribution of the initial materials and the obtained biocatalytic systems. The SEM images of the ZIF-8 material (Figure 3a) show the crystalline structure of the support, which has overlapping crystals with an average particle size of about 290 nm. It is possible to observe distinct clusters of enzymes on the surface of the support after immobilization (Figure 3b), which is confirmed by the increased average particle size of the resulting biocatalytic system (approximately 440 nm). ZIF-8 is an attractive material for immobilizing enzymes, as demonstrated in a study by Stanišić et al. [40], which showed the successful immobilization of HRP on this support mainly due to well-fitted particles. Figure 3c presents the morphology of pure MWCNTs. The presence of bundles of intertwined tubes with inhomogeneous lengths of a few micrometers is characteristic of this carrier. In the MWNCT material, its morphology allows enzymes to be efficiently immobilized since there are many open spaces where the enzymes can be localized onto and into the support’s structure. It is possible to notice in Figure 3d that a thin layer of enzyme was formed on the surface of the support after HRP immobilization. A similar distribution of the enzyme on the surface of MWCNTs is shown in studies conducted by Duarte et al. [41], confirming our assumptions on MWCNTs’ suitability as supports for HRP.

Figure 3e presents an SEM image of the pristine chitosan-coated magnetic nanoparticles doped with selenium ions. Chitosan is characterized by its smooth, non-porous structure, which is made up of crystals and microfibers of varying sizes. An analysis of the resulting biocatalytic system (Figure 3f) shows that HRP enzymes are present both as agglomerates and as a single molecules. Figure 3g shows SEM images of the magnetic nanoparticles, which are characterized by a homogeneous distribution and spherical shape. The particle size of the used MNPs is quite narrow and ranges between 141 and 396 nm, while the average size is about 280 nm. As a result of dipole–dipole interactions between the Fe_3_O_4_ nanoparticles, this material also exhibits an agglomeration of these particles. After the immobilization of the HRP enzyme, there are no significant changes in the morphology of the support (Figure 3h), which is consistent with the observations of Gong et al. [42]. Note, however, that particles bigger than 100 nm were identified in the material upon immobilization, clearly indicating HRP deposition onto the surface of the MNPs. A morphological analysis of graphene oxide’s structure (Figure 3i) shows its layered structure. The layers have irregular shapes and different sizes (particle sizes between 458 and 1718 nm). Also typical for this support is the presence of pronounced distortions, which form rough graphene oxide surfaces and are due to the presence of oxygen groups in its structure. Figure 3j provides information on the morphology of the biocatalytic system formed after the immobilization of HRP on graphene oxide. It shows the deposition of enzyme layers on the surface of graphene oxide sheets and the presence of larger agglomerates of the enzyme, supported also by the results of particle size distribution, which showed an increase in the average particle size distribution upon immobilization. These data stay in agreement with the results presented by Besharati et al. [43] and Devaraj et al. [24]. However, HRP immobilization using graphene oxide is not widely presented in the literature.

The scanning electron microscopy analysis provides information regarding the structure and morphology of the materials, indicating that all the materials are suitable for use as enzyme carriers. In addition, the immobilization process does not affect the structure of the materials, which allows them to retain their specific characteristics even after enzyme deposition, making the produced systems stable and resistant [44,45]. However, due to enzyme binding, in most cases, an increase in the particle size of the support materials occurred, which confirms efficient HRP immobilization.

The energy-dispersive X-ray microanalysis (EDS) provided valuable information on the elemental composition of the obtained materials as well as the resulting biocatalytic systems after HRP immobilization (Table 2). For example, the EDS results can be used to verify the chemical composition of the MNPs, whose high iron content (above 60%) confirms the correct synthesis process. The presence of selenium in the CH-M-Se material also confirms the incorporation of selenium nanoparticles and the formation of the desired hybrid material. Furthermore, the immobilization of the enzymes increases the oxygen content of all the resulting biocatalytic systems due to the presence of many oxygen groups in the enzyme structure. In materials with immobilized enzymes, there is also an increase in nitrogen content. The work of Işık [46] noted a significant increase in oxygen and nitrogen content after immobilizing chitosan beads. Similarly, Zdarta et al. [47] observed that enzyme deposition led to an increase in carbon and oxygen content, as well as the appearance of nitrogen due to the presence of an enzyme immobilized on the carrier.

**Figure 4:**
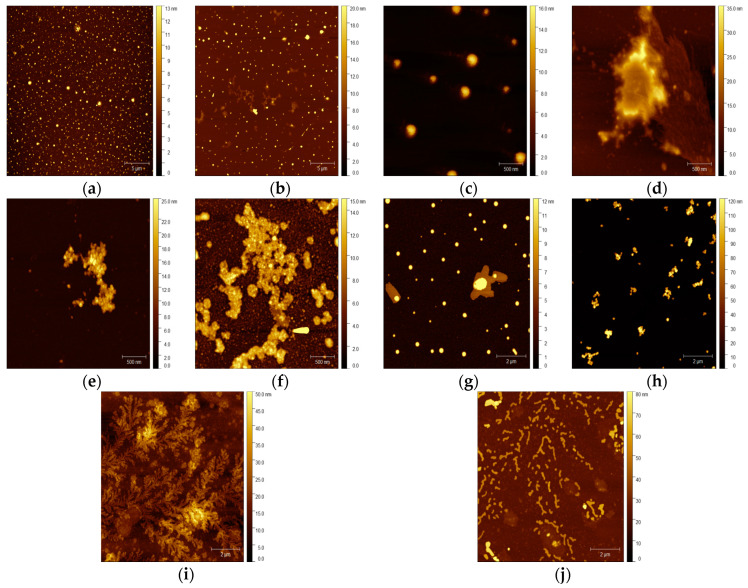
AFM images: (**a**) pristine ZIF-8 carrier; (**b**) ZIF-8 carrier after HRP immobilization; (**c**) pristine MWCNT material; (**d**) MWCNTs after HRP immobilization; (**e**) CH-M-Se carrier; (**f**) CH-M-Se carrier after HRP immobilization; (**g**) MNPs; (**h**) MNPs after HRP immobilization; (**i**) graphene oxide material; and (**j**) graphene oxide after HRP immobilization.

ZIF-8 is characterized by its easy preparation and biocompatibility and has been previously demonstrated to be an efficient carrier for enzyme immobilization. Within the performed experiments, small particles with visible crystal structures of the ZIF-8 material were visualized using the atomic force microscopy (AFM) technique (Figure 4a). This method enables a non-destructive analysis of the carrier surface topography and the characterization of immobilized enzyme molecules on the carriers’ surfaces [48]. According to the photographs of the ZIF-8 surface after HRP immobilization, this process resulted in a rounding of the carrier particle shape and a smoothing of their edges. Visible changes in the particle morphology and the increased size of the carrier particle agglomerates prove the sufficient enzyme immobilization on the tested material. It is worth noting that the ZIF-8 material has been tested for the efficient immobilization of various enzymes, e.g., pepsin, as performed by Yang et al. [49]. The next tested material, multi-walled carbon nanotubes, is an important class of new materials with numerous useful properties, including suitability for enzyme immobilization. The performed experiments resulted in the deposition of the enzyme molecules on the surface of the nanotubes as well as inside the tubes, which was compared to the pure carrier (Figure 4b,d).

The observed increases in the nanotube size align with the findings of Rastian et al. [50], who employed MWCNTs for lipase immobilization. They also applied MWCNT surface functionalization for better incorporation of the enzyme, an approach that was tested in our study in the case of the selenium-functionalized material Ch-M-Se. The presence of these ions increases the stability of the biocatalyst, resulting in better properties for the created system. Comparing the pictures of the pure and enzyme-loaded Ch-M-Se carriers (Figure 4e,f), one can see changes in the carrier surface topography after immobilization, as well as a higher tendency to form aggregates. This was also confirmed by the particle size investigation of the support materials before and after HRP immobilization. Another class of materials with possible applications in enzyme immobilization are magnetic nanoparticles, which, due to their magnetic properties, enable the easy separation of the carrier from the reaction mixture [51]. This feature makes them a promising tool for industrial bioprocess applications. Our study confirmed changes in the MNPs’ behavior after the immobilization (Figure 4h), showing a higher preference for self-association. The increase in the size of the tested MNPs is in line with reports by Mendes et al. [52] and Arredondo et al. [53], who tested various magnetic carriers in tyrosinase immobilization. Finally, graphene oxide (GO), a cheap and easy-to-process material that can be viewed as a single atomic layer of graphite with various oxygen-containing functionalities, was applied to HRP immobilization. Comparing the topography of the native and HRP-immobilized graphene oxide surfaces, a significant increase in the height of the surface can be seen, which confirms the effective deposition of the enzyme on the surface of the material. Regarding the analysis presented by Zhang et al. [54], the determinants of the conformation of the immobilized enzyme are the interactions between the functional groups of GO and HRP. The presented results confirm that AFM is a powerful tool for analyzing material topography modifications after enzyme immobilization. In this case, this technique allows for the confirmation of effective HRP immobilization on matrices with various properties and applications, supporting results from other experiments.

### 2.2. Biocatalytic Characterization of the Produced Systems

The immobilization process was carried out under the most favorable conditions, and to confirm the effectiveness of the produced biocatalytic systems, the process yield, the amount of immobilized enzyme, and the retained catalytic activity were determined (Table 3). The process of HRP immobilization on all the tested supports occurred with an efficiency of at least 69%, regardless of the process parameters used, with the maximum at around 85–90%. The highest efficiencies were recorded for the processes using MWCNTs and CH-M-Se materials, which were 89.2% and 84.9%, respectively. Further, the amount of immobilized enzyme was examined to determine its loading and possible distribution within the support structures. The process occurs most efficiently at the initial stage, when the availability of active sites on the surface of the carrier is the highest. The MWCNTs and CH-M-Se also turned out to be matrices that allowed for the attachment of as much as 44.6 mg and 42.5 mg per 1 g of carrier, respectively. These nanomaterials exhibit a unique combination of stiffness and strength compared to other fibrous materials and offer ideal properties such as resistance to weight transfer and large surface areas. However, the amount of immobilized enzyme is not everything, as it is crucial for the production of effective biocatalytic systems that the immobilized protein maintains high activity. Analyzing the data in Table 3, it can be concluded that the produced systems retain high activity, which is about 80%. The exception is graphene, which was characterized by the lowest activity of immobilized HRP (62%). In contrast, CH-M-Se distinguished itself from the other materials by retaining catalytic activity exceeding 90%. Selenium’s favorable properties, such as high biological activity, non-toxicity, and good bioavailability, have made the element increasingly popular. Moreover, the combination of chitosan and magnetic nanoparticles in the structure of this hybrid support facilitates easy substrate/product flow and reduces possible diffusional limitations. For example, Nonsuwan et al. [55] used selenium to develop organic–inorganic hybrid pullulan/SeNPs microflowers, which exhibited an excellent enzyme adsorption of 142.2 mg/g. Taking all parameters into account, the process of HRP immobilization on the proposed materials can be considered efficient and allows for the immobilization of significant amounts of biocatalyst on the surface of the carrier, supported by high activity recovery. CH-M-Se is the carrier with the most favorable properties for binding the HRP enzyme. Chitosan-based nanofibers are characterized by increased porosity, excellent mechanical properties, and a high surface-to-volume ratio, and the addition of selenium gives these carriers unique properties that can improve enzyme immobilization efficiency and enzyme stability.

**Figure 5:**
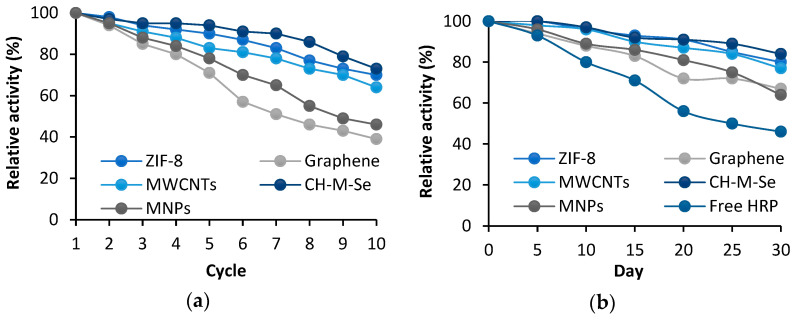
Reusability (**a**) and storage stability (**b**) of HRP immobilized using tested support materials. The results are the mean values of three repeated experiments with an error value of less than 5%.

An undoubtedly distinguishing feature of the immobilized enzymes is the improvement in their stability and potential for reuse. To determine the reusability of the produced biosystems, 10 consecutive reaction cycles were carried out, the results of which are summarized in Figure 5a. Analyzing the data presented, it can be seen that 100% of activity was retained only after the first cycle, and this gradually decreased with each subsequent cycle. All the materials were good matrices for HRP immobilization, as they retained their activity after 10 cycles, but clear percentage differences were noted. The lowest activity was retained by graphene and the MNPs, at levels of 39% and 46%, respectively, while the highest potential was shown by CH-M-Se, retaining its activity at 73%. Such a high percentage result may indicate the formation of stable bonds between the enzyme and the surface of the carrier, which effectively prevents the leaching of HRP. The addition of selenium further increases the stability of the enzyme and makes the system more resistant to varying process conditions and repeated use, as the selenium ions show a positive effect on HRP activity. Finally, the addition of magnetic nanoparticles as a part of the hybrid support strongly enhances the operability of the immobilized system because the simple separation of the system from the post-reaction mixture is possible, which reduces the number of operational stages negatively affecting the enzyme stability. The storage stability of the biocatalytic systems and the free enzyme was also investigated. The results of these tests are shown in Figure 5b. A decrease in the activity of the free HRP was observed from the first days of testing, and this eventually led to the preservation of only about 46% of the activity after 30 days. The loss of activity over one month for the immobilized enzyme was significantly less. All the support materials tested significantly improved the retained activity, and these results confirm the benefits of immobilization in increasing enzyme stability. The best results were achieved for the CH-M-Se and ZIF-8 materials, as the HRP immobilized on their surfaces retained activity at 84% and 80%, respectively, after 30 days of storage. Similar results were also observed by Xie et al. [56], who observed that after 60 days of storage, the activity of HRP immobilized using the multi-arm magnetic composite Fe_3_O_4_@PAA-6-arm-PEG-NH_2_ was 71% higher than that of the free enzyme, which also confirms the protective properties of the carrier for HRP. All the examples cited confirm the validity of using carriers as scaffolds that stabilize the structure of enzymes and exhibit protective functions against catalytic proteins, thus limiting conformational changes of proteins during storage. Table 4 presents a comparison of results from previous studies with the results presented in this study to highlight the importance of the obtained results. It should be added that, compared to traditional enzyme carriers, the use of Ch-M-Se and MWCNT materials characterized by unique properties translates into effective enzyme immobilization and improved stability. The use of these matrices ensures the preservation of high enzymatic activity and provides the possibility of reusable systems, which affects the cost-effectiveness of the process. The structure of MWCNTs additionally provides a large enzyme contact area, which increases the efficiency of enzymatic reactions, while Ch-M-Se materials exhibit excellent biocompatibility, which promotes the long-term stability of biocatalytic systems. Despite the higher cost of Ch-M-Se material synthesis as compared to other simple and commercially available support materials the significant benefits of using it as a carrier for enzyme immobilization make the resulting biocatalytic systems economically viable solutions at the laboratory scale. Nevertheless, deeper studies on the cost-effectiveness of the entire biocatalytic process supported by the immobilized HRP are required, which will facilitate the translation of the proposed system onto a larger scale.

**Figure 6:**
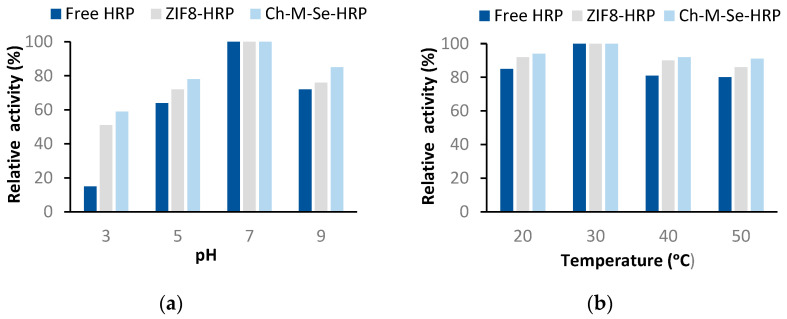
Effect of pH (**a**) and temperature (**b**) on the relative activity of free and immobilized HRP on support materials. The results are the mean values of three repeated experiments with an error value of less than 5%.

After the evaluating the stability potential of the immobilized HRP and characterizing the resulting biocatalytic systems, it is also important to determine its activity changes under different process conditions. In this section, we decided to present only systems obtained using the chitosan-based hybrid material and ZIF-8 materials, because when using these supports, the highest activity recovery and stability were attained. Analyzing the effect of pH and temperature on the retained catalytic activity of the systems (Figure 6), one can see the greater tolerance of the immobilized enzyme to changing conditions. The only exception where identical profiles and the highest activity were obtained for the free and immobilized HRP is at 30 °C and a pH of 7, hence, at the optimal process conditions. Outside these conditions, the free HRP showed lower activity and was found to be more prone to inactivation. More favorable results for the immobilized enzyme were achieved when using the Ch-M-Se material as a support. This system showed over 80% activity over a pH range of 5 to 9 and over the whole analyzed temperature range, clearly showing the most stabilizing and protective effect of this support on the HRP structure. This is related to the fact that chitosan contains amine and hydroxyl groups in its structure that support stable enzyme binding, even including multipoint attachment, which makes the enzyme structure more rigid and stable under harsh process conditions.

## 3. Materials and Methods

### 3.1. Materials and Reagents

Graphene, multi-walled carbon nanotubes, zeolitic imidazole framework, magnetic nanoparticles, and chitosan-magnetic nanoparticles doped with selenium ions hybrid material were synthesized by the members of the Division of Chemical Technology at the Faculty of Chemical Technology, Poznan University of Technology and were used as delivered, without any further purification. Horseradish peroxidase (EC 1.11.1.7) (HRP), hydrogen peroxide solution (30%), 50 mM phosphate buffer at pH 7, Bradford reagent, and 2,2′-azino-bis(3-ehylbenzothiazoline-6-sulfonic acid) diammonium salt (ABTS) were delivered by Sigma-Aldrich (St. Louis, MO, USA).

### 3.2. HRP Immobilization

The immobilization of horseradish peroxidase (HRP) using various materials was carried out as follows. Precisely, 100 mg of the selected carrier material was weighed, and an HRP solution with a concentration of 0.5 mg/mL (10 mL) was added. The process lasted for 24 h at room temperature and pH 7, and the samples with the carrier and enzyme solution were mixed at a speed of 300 rpm in a thermomixer. After immobilization, the samples were centrifuged. The resulting precipitate was allowed to dry at room temperature, while the solution above the sediment was analyzed to determine the content of the unbound enzyme and calculate the process efficiency.

### 3.3. Physicochemical Characterization of the Produced Systems

The morphology of the used materials before and after immobilization was determined based on SEM images (EVO40, Zeiss, Oberkochen, Germany) after being coated by gold (Balzers PV205P, Balzers, Switzerland). An energy-dispersive X-ray microanalysis (EDS) was carried out using the EDS analyzer contained in the scanning electron microscope (Tescan machine (Tescan, Switzerland) with Gamma-Tec tooling from Priceton Inc. ((Princeton, NJ, USA). Before the analysis, the samples were attached to the base with a carbon paste or tape. The FTIR spectra of the samples were made using a Vertex 70 spectrometer (Bruker, Bremen, Germany). The samples were analyzed in the form of KBr pellets in the wavenumber range 4000–400 cm^−1^ at a resolution of 0.5 cm^−1^ with 64 scans per sample. An AFM (Park NX10, Park Systems Corp., Suwon, Korea) was implemented in the experiments to determine changes in the topography of the support surface before and after immobilization. Topography measurements were performed in non-contact mode with an All-in-One cantilever, type D (BudgetSensors, Sofia, Bulgaria), with a nominal resonance frequency of 350 kHz and a nominal force constant of 40 N/m. The scan size was set to 10 × 10 μm^2^ with a sampling of 512 lines for each image and a scan rate of 0.4–0.5 Hz. For each sample, at least three measurements were performed at different positions. The measurements were performed in air at room temperature. The results of the imaging were further investigated using Gwyddion v 3.05 version open-source software. Particle size distributions were determined using a Zetasizer Nano ZS (Malvern Instruments Ltd., Malvern UK), enabling measurements of particle diameters in the range 0.6–6000 nm based on a noninvasive backscattering technique, NIBS. The measurement involves transmitting a red laser beam with a wavelength of 663 nm through the material. A thermogravimetric analysis (TGA) of the materials before and after immobilization was performed using a Jupiter STA 449F3 apparatus (Netzsch, Selb, Germany). Measurements were carried out over the temperature range of 25–1000 °C under nitrogen flow (10 mL/min) at a heating rate of 10 °C/min, with an initial 10 mg mass of the sample.

### 3.4. Biocatalytic Characterization of the Produced Systems

The activity and stability of the free and immobilized HRP were examined based on a model reaction using ABTS as a substrate by measuring the rate of formation of ABTS*. Briefly, 10 mg of free enzyme or an appropriate amount of the biocatalytic system containing 10 mg of immobilized HRP was added to the solution containing 15 mL of 100 mM ABTS solution in 50 mM phosphate buffer at pH 7.0 and 1 mL of 5 mM hydrogen peroxide. The process was carried out for 10 min at 30 °C, and the progress of the oxidation reaction was measured with a Jasco V-750 (Tokyo, Japan) spectrophotometer at λ = 420 nm. One unit of free and immobilized HRP activity was defined as the amount of enzyme that produces 1 mM of ABTS* per minute under assay conditions.

The amount of immobilized enzymes on tested support materials was determined according to the Bradford method by considering the mass of the immobilized enzyme and the mass of the support material. After immobilization, the solutions were mixed with Bradford reagent in a 0.5 mL:0.5 mL ratio. Following 10 min of incubation, the absorbance was measured at 595 nm, and the HRP concentration was determined using a standard curve of BSA. The immobilization yield and activity recovery were calculated according to Equations (1) and (2):

(1) $$Immobilization\;yield\;\left(\%\right)=\frac{A_{i}-A_{f}}{A_{i}}\times 100\%\;$$

(2) $$Activity\;recovery\;\left(\%\right)=\frac{A_{t}}{A_{i}}\times 100\%$$

where *A_i_* denotes the initial activity of the HRP used for immobilization, *A_f_* denotes the total activity of the enzyme in the supernatant, and *A_t_* denotes the activity of the immobilized HRP. The activities of the supernatant and reference samples were determined under the standard conditions based on the above-presented reaction.

The effect of pH on the activity of the immobilized HRP was examined according to the above-mentioned ABTS reaction at 30 °C over a pH range of 3 to 9, whereas the effect of temperature was examined over a temperature range of 20 to 50 °C at pH 7.

The reusability of the produced system was examined for over 10 consecutive catalytic cycles using ABTS as a model substrate. The ABTS relative conversion efficiency was calculated based on the final concentration of ABTS free radicals after the process. After each cycle, the system tested was washed with 50 mM of phosphate buffer at pH 7.0 and placed in a fresh substrate solution. The storage stability of the free and immobilized HRP stored at 4 °C in phosphate buffer (pH 7.0) was tested for over 30 days. The relative activity was determined over time based on the ABTS model oxidation reaction. For analyzing the storage stability and reusability, the initial activity was defined as 100% activity. All measurements were conducted in triplicate, and the results are presented as mean value ± standard deviation.

## 4. Conclusions

This study synthesized and tested novel biocatalytic systems based on various hybrid and single materials as successful platforms for enzyme deposition. The morphological analysis, stability, and activity tests were performed to fully characterize the successful biocatalysts’ deposition and their catalytic activity. Based on the morphological analysis of the obtained biocatalytic samples, such as the FTIR, TGA, and AFM images, the synthesis of the materials and immobilization were proven. A further indication of the success of the immobilization process was the increase in particle size following the immobilization of the enzyme. The analyses carried out using UV-Vis spectroscopy confirmed the ability to apply significant amounts of the enzyme to the surface of the carriers. The immobilization of HRP on all the tested supports occurred with an efficiency of at least 69%, and for the MWCNT and CH-M-Se materials, the efficiency reached up to 90%. The obtained results showed that the retained activity was high at around 80%, reaching over 90% for CH-M-Se. Considering the results of all the above analyses, it was concluded that CH-M-Se was the carrier with the most favorable properties for binding the HRP enzyme. The addition of selenium gives these nanomaterials unique properties that translate into efficient enzyme immobilization. Also, the storage stability tests and analyses of the reusability of the biocatalytic systems showed the most favorable results for the enzyme immobilized on CH-M-Se, which clearly confirms the suitability of using these nanomaterials as scaffolds that stabilize the structure of HRPs and show protective functions against catalytic proteins. The presented studies demonstrate the applicability of the proposed hybrid and single materials as platforms for the successful synthesis of biocatalytic systems. The developed systems exhibit a broad spectrum of potential applications due to their high catalytic activity and reusability, hence promoting the growth of the green and sustainable technologies industry.

## Figures and Tables

**Table 1:** The average particle size of the support materials before and after HRP immobilization.

Type of Material	Sample	Average Particle Size (nm)	Particle Size Range (nm)
Pristine materials	ZIF-8	287	105–615
	graphene	630	458–1718
	MWCNTs	62	5.6–141
	CH-M-Se	832	190–2801
	MNPs	283	141–396
Materials after HRP immobilization	ZIF-8	443	255–825
	graphene	806	615–1281
	MWCNTs	112	17–198
	CH-M-Se	973	295–2969
	MNPs	372	37–643

**Table 2:** Elemental composition of carriers before and after immobilization.

Samples		Content (%)						
	C	N	O	S	Mn	Fe	Zn	Se
ZIF-8	42.04	6.49	30.27	-	-	0.01	21.16	-
ZIF-8 + HRP	42.19	10.78	30.78	-	0.02	0.03	16.18	-
graphene	97.38	-	2.17	0.25	0.01	0.19	0.01	-
graphene + HRP	82.32	-	17.19	0.21	0.03	0.22	0.02	-
MWCNTs	91.83	-	7.92	0.02	-	0.23	-	-
MWCNTs + HRP	85.11	-	14.73	0.01	-	0.15	-	-
CH-M-Se	15.99	0.13	32.45	0.10	26.87	2.37	0.01	22.08
CH-M-Se + HRP	12.72	0.15	33.25	0.05	29.75	3.09	-	20.98
MNPs	10.24	-	29.21	-	0.03	60.51	-	-
MNPs + HRP	24.79	-	32.12	0.02	0.03	43.02	0.03	-

**Table 3:** Results of immobilization yield, amount of immobilized enzyme, and activity recovery.

Sample	Immobilization Yield (%)	Amount of Immobilized Enzyme (mg/g)	Activity Recovery (%)
ZIF-8	69.8	34.9	81.2
graphene	84.6	42.3	62.7
MWCNTs	89.2	44.6	87.3
CH-M-Se	84.9	42.5	91.2
MNPs	74.3	37.1	83.8

**Table 4:** Comparison of the HRP immobilization results obtained with those obtained from previous studies.

Carrier	Immobilization Method	Immobilization Efficiency (%)	Activity Recovery (%)	Reusability	Optimum pH and Temperature	Reference
MNPs	adsorption	7	100	55% after 10 cycles	7.5 50 °C	[15]
MNPs	adsorption	74.3	83	46% after 10 cycles	7 30 °C	present study
graphene	covalent bonding	30	90	70% after 10 cycles	7 50 °C	[43]
graphene	adsorption	84.6	61	39% after 10 cycles	7 30 °C	present study
ZIF-8	encapsulation	98.4	89	>80% after 6 cycles	6.5 70 °C	[57]
ZIF-8	adsorption	69.8	81	70% after 10 cycles	7 30 °C	present study
MWCNTs	covalent bonding	81	80	not available	7 30 °C	[58]
MWCNTs	adsorption	89.2	87	70% after 10 cycles	7 30 °C	present study
CH-M-Se	adsorption	84.9	91	73% after 10 cycles	7 30 °C	present study

## Data Availability

The raw data supporting the conclusions of this article will be made available by the authors on request.

## Abbreviations

ABTS	(2,2′-azino-bis(3-ethylbenzothiazoline-6-sulfonic acid))
AFM	Atomic force microscopy
APTES	(3-Aminopropyl)triethoxysilane
Ch-M-Se	Chitosan-magnetic nanoparticles doped with selenium ions hybrid material
EDS	Energy-dispersive X-ray microanalysis
FTIR	Fourier-transform infrared spectroscopy
GO	Graphene oxide
HRP	Horseradish peroxidase
MNPs	Magnetic nanoparticles
MWCNTs	Multi-walled carbon nanotubes
SEM	Scanning electron microscopy
TGA	Thermogravimetric analysis
TiO_2_	Titanium dioxide
ZIF-8	Zeolitic imidazole framework
